# Winter amplification of the European Little Ice Age cooling by the subpolar gyre

**DOI:** 10.1038/s41598-017-07969-0

**Published:** 2017-08-30

**Authors:** Eduardo Moreno-Chamarro, Davide Zanchettin, Katja Lohmann, Jürg Luterbacher, Johann H. Jungclaus

**Affiliations:** 1 Max Planck Institute for Meteorology, Hamburg, Germany; 2 International Max Planck Research School on Earth System Modeling, Hamburg, Germany; 3 Department of Earth, Atmospheric and Planetary Sciences, Massachusetts Institute of Technology, Cambridge, USA; 4 University Ca’Foscari of Venice, Department of Environmental Sciences, Informatics and Statistics, Mestre, Italy; 5 Department of Geography, Justus Liebig University of Giessen, Giessen, Germany; 6 Center of International Development and Environmental Research, Justus Liebig University of Giessen, Giessen, Germany

## Abstract

Climate reconstructions reveal a strong winter amplification of the cooling over central and northern continental Europe during the Little Ice Age period (LIA, here defined as c. 16th–18th centuries) via persistent, blocked atmospheric conditions. Although various potential drivers have been suggested to explain the LIA cooling, no coherent mechanism has yet been proposed for this seasonal contrast. Here we demonstrate that such exceptional wintertime conditions arose from sea ice expansion and reduced ocean heat losses in the Nordic and Barents seas, driven by a multicentennial reduction in the northward heat transport by the subpolar gyre (SPG). However, these anomalous oceanic conditions were largely decoupled from the European atmospheric variability in summer. Our novel dynamical explanation is derived from analysis of an ensemble of last millennium climate simulations, and is supported by reconstructions of European temperatures and atmospheric circulation variability and North Atlantic/Arctic paleoceanographic conditions. We conclude that SPG-related internal climate feedbacks were responsible for the winter amplification of the European LIA cooling. Thus, characterization of SPG dynamics is essential for understanding multicentennial variations of the seasonal cycle in the European/North Atlantic sector.

## Introduction

Recent paleoclimate evidence indicates that the onset, spatial extent, and magnitude of the cold period between the 16th and 18th centuries—known as Little Ice Age (LIA)—varied distinctly across regions^1^. In particular, the European continent experienced a maximum summer cooling of about 1 K in the early 17th century compared to the mean temperature in the late 20th century^1, 2^. However, the magnitude of the LIA cooling in Europe also varied across seasons. Early instrumental data and documentary-based local temperature reconstructions demonstrate a notable increase in the duration and severity of the winter season^3–8^, suggesting that decadal-scale temperature anomalies could have been more than 2–3 K colder in the early 17th century than in the late 20th century^9, 10^. The consequent shortening of the growing season by several weeks deeply affected societies throughout the continent as it caused widespread crop failure, famine, and population decline in some regions^4, 11^. On the other hand, summer seasons were characterized by fluctuations between normal and extremely hot or cold conditions^1–8^, including, for example, the “year without a summer 1816 CE” that was clearly related to the eruption of Mount Tambora in April 1815. Yet, the reason behind this seasonal contrast has remained largely unexplored—to some extent because most climate proxies reflect boreal summer conditions^1, 2^.

Early studies attributed the European LIA cooling to periods of low solar activity, such as the Maunder Minimum^12^. Later studies then suggested a more important role of strong volcanic eruptions in the onset of the LIA^13–15^, through amplifying internal climate feedbacks, involving a reduced Atlantic meridional overturning circulation (AMOC)^15, 16^, and/or a varying (albeit mainly negative) phase of the North Atlantic Oscillation (NAO)^17^. However, such long-lasting anomalous states of the AMOC and NAO and, by extension, their role in the development of the LIA cooling in Europe have found no support in recent AMOC^18^ and NAO^19^ reconstructions. Therefore, the main drivers of the European LIA cooling and its seasonal features remain elusive.

**Figure 1:**
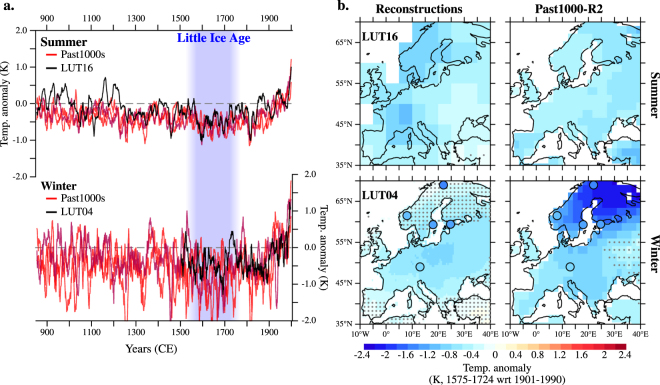
Simulated and reconstructed European temperatures. (**a**) Simulated (red) and reconstructed (black) European temperature anomalies in summer (top) and winter (bottom), calculated as the spatial average over the land-only region between 35°N–70°N and 10°W–40°E (shown in (**b**)) and smoothed with an 11-year running mean. Past1000-R2 is highlighted in dark red (see Methods). (**b**) Land temperature anomalies for the period 1575–1724 CE (shading in (**a**)) reconstructed (left) and simulated in Past1000-R2 (right) in summer (top) and winter (bottom). Stippling masks statistically non-significant anomalies at the 5% level. LUT16 and LUT04 are for refs 2 and 3 respectively. Circles indicate the location of independent winter temperature reconstructions (see Methods), with their color accounting for the corresponding temperature anomaly (i.e., with the same color scale as for the background temperature field). Anomalies are calculated with respect to 1901–1990 CE. Maps were generated in *Pyferret* v. 7.0. (Information is available at http://ferret.pmel.noaa.gov/Ferret/).

Here, we describe the physical mechanism underlying the seasonal asymmetry in the European LIA cooling via an ensemble of three climate simulations of the past millennium (past1000-R1/-R2/-R3; see Methods) performed with the Max Planck Institute Earth System Model (MPI-ESM; see Methods). The simulated climatic variations in the ensemble are consistent with the most recent and comprehensive reconstructions of European temperature variability over the Common Era (LUT16 and LUT04 for refs 2 and 3, respectively; see Methods). With respect to the average temperature of the period 1901–1990 CE, the reconstructed continental-scale European temperature (Fig. 1a) shows persistent cold conditions throughout the 16th–18th centuries, both in summer (June–August, JJA) and winter (December–February, DJF). The magnitude of the winter anomaly is approximately twice of that in summer, which illustrates the above-mentioned increase in winter duration and severity recorded during the LIA.

The continental-scale summer temperatures in the three model simulations show statistically significant (p < 0.05) correlation with the LUT16 reconstruction over the past millennium on decadal and longer time scales (Suppl. Table 1), and especially during the coldest LIA centuries after 1500 CE, for which model and data exhibit strong co-variability. In winter, variability is more pronounced, and the agreement between the simulations and the reconstructions is thereby lower, with statistically non-significant (p > 0.05) correlation coefficients at these time scales (Suppl. Table 1). Nonetheless, correlation coefficients across model simulations show similar values to those between the simulations and the reconstructions, both in winter and summer, so that the agreement with the reconstructions lies largely within the intra-ensemble range in each season. This result suggests that the reconstructions cannot be statistically distinguished from an equivalent, additional model realization. Thus, both reconstructions and simulations provide a similar picture of the LIA cooling, with a prominent winter amplification. Accordingly, hereafter we focus our analysis on one model realization, Past1000-R2 (shown in darker red in Fig. 1a), because it agrees the best with the summer and winter reconstructions (Suppl. Table 1); nevertheless, results can be extended to the other two Past1000 simulations.

In summer, variations in the European continental-scale temperature on decadal and longer time scales trace closely those variations in the mean temperature of the entire Northern Hemisphere in both the simulations and the reconstructions (Suppl. Figure 1). Thus, temperature excursions such as the general LIA cooling or the 20th-century warming that can mainly be attributed to external forcings^13, 20^ feature similar magnitude, timing, and duration on hemispheric and regional scales. Therefore, European summer temperature variability on decadal and longer time scales is mostly driven by direct radiative responses to externally forced imbalances in the Earth’s energy budget. In contrast, variability in the European winter temperature shows much larger amplitude than that of the Northern-hemispheric mean (Suppl. Figure 1). This finding suggests a strong effect of specific regional dynamical features on the evolution of the European climate in winter, which results in the amplification of the LIA cooling.

Such a regional effect is particularly relevant in Scandinavia and central Europe, where the reconstructed and simulated winter cooling is strongly amplified during the LIA’s coldest period at the end of the 17th century (Fig. 1b). In contrast, during the same period, summer temperature anomalies are more homogeneously distributed over the continent. Note, however, that the reconstruction techniques differ considerably between LUT16 and LUT04 (see Methods), with the latter underestimating the low-frequency temperature variability^21^. The magnitude of the reconstructed LIA winter cooling is therefore smaller than that in the simulation, albeit it is almost twice as large as the LIA cooling in the European summer reconstruction for the same period in LUT04 (Suppl. Figure 2). Other independent reconstructions of winter temperature (colored circles in Fig. 1b) exhibit larger LIA cooling in northern Europe than the one of the central European record; this anomalous cooling pattern is consistent with the simulated one.

**Figure 2:**
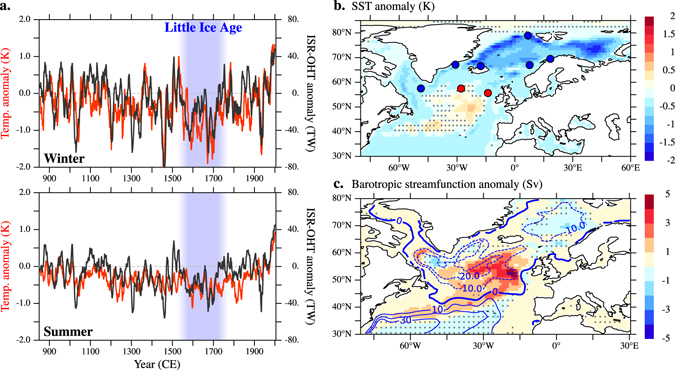
Linking the European LIA cooling to a SPG weakening. In Past1000-R2: (**a**) ISR-OHT and continental-scale European temperature anomalies in winter (top) and summer (bottom), smoothed with an 11-year running mean (with respect to 1901–1990 CE). (**b**,**c**) Anomalies in the annual mean sea-surface temperature (SST) and barotropic streamfunction (shading, with PiControl climatology in contours; see Methods) for the period 1575–1724 CE (shading in (**a**)) with respect to the PiControl climatology. Note that positive anomalies of the barotropic streamfunction within the SPG region indicate a weakening of the anticlockwise (i.e., negative) flow. Stippling masks statistically non-significant anomalies at the 5% level. Red/blue circles (**b**) indicate relatively warm/cold upper-ocean temperatures during the LIA from a collection of available proxies (see Methods). Maps were generated in *Pyferret* v. 7.0. (Information is available at http://ferret.pmel.noaa.gov/Ferret/).

**Figure 3:**
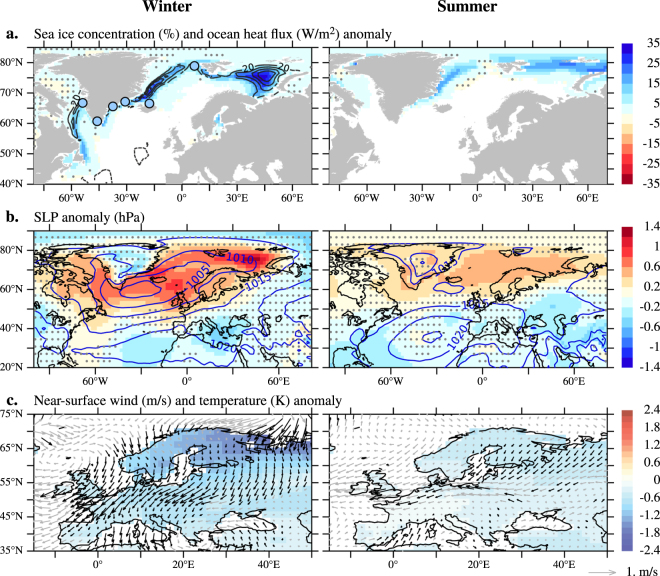
Seasonal asymmetries in climate changes during the LIA. In Past1000-R2, anomalies for the period 1575–1724 CE—calculated as in Fig. 2b—in (**a**) sea ice concentration (shading) and ocean surface downward heat flux (contours at intervals of 20 W/m^2^) (**b**) SLP (shading, with PiControl climatology in contours) and (**c**) near-surface wind (arrows) and temperature (shading), in winter (left) and summer (right). Sea ice concentration is in late winter (March) and late summer (September), when it reaches its climatological maximum and minimum extension respectively. Stippling or gray arrows masks statistically non-significant anomalies at the 5% level. Blue circles ((**a**) left) indicate expanded sea ice conditions during the LIA from a collection of available proxies (see Methods). Maps were generated in *Pyferret* v. 7.0. (Information is available at http://ferret.pmel.noaa.gov/Ferret/).

In the model, European cold conditions during the LIA are connected in both winter and summer with a multicentennial reduction in the northward oceanic heat transport across the Iceland–Scotland Ridge (ISR-OHT; Fig. 2a), which causes widespread upper-ocean cooling in the Nordic and Barents seas (Fig. 2b; the oceanographic components mentioned here are indicated in Suppl. Figure 3b). The ISR-OHT reduction is more relevant for the European climate in winter than in summer due to seasonal differences in the atmospheric response to changes in sea ice cover and surface heat flux related to ISR-OHT variability (Suppl. Figure 4). In winter, upper-ocean cooling driven by a weak ISR-OHT (Fig. 2b and Suppl. Figure 4a) enables extensive sea ice growth in the Nordic and Barents seas (Fig. 3a and Suppl. Figure 4c). In turn, the sea ice expansion dampens the ocean heat release to the atmosphere (Fig. 3a and Suppl. Figure 4c), which thereby cools down (Suppl. Figure 4b). The near-surface atmospheric cooling induces more stable wintertime atmospheric conditions, as illustrated by the development of positive sea-level pressure (SLP) anomalies over the Barents Sea throughout the 16th–18th centuries (Fig. 3b). However, the response of the atmosphere is not locally confined to this region: positive SLP anomalies also extend to the subpolar North Atlantic and northern Europe and, hence, induce substantial, long-lasting changes in the atmospheric circulation over the continent (Fig. 3c). Such a connection between a reduced ISR-OHT and anomalously high low-level pressures over the Barents Sea is consistent with previous studies using modern observations or idealized climate simulations (see, for example, ref. 22 and references therein).

Associated with the positive SLP anomalies, anomalous wintertime near-surface north-northeasterlies over northern, central, and eastern Europe advect cold polar air masses from the Barents Sea/Arctic region and Western Russia toward the continent (Fig. 3c). Likewise, anomalous easterlies over western Europe divert the westward flow of warm, humid air masses of Atlantic origin away from the continent (Fig. 3c). The more stable atmospheric conditions during the LIA also increase the frequency of wintertime blocking events, especially in Scandinavia (Suppl. Figure 5). In Europe, persistent blocked atmospheric conditions are associated with weather extremes, such as cold spells or floods^23, 24^. Synoptic patterns during blocking events can reduce near-surface temperature below freezing for weeks or months due to anomalous advective heat transport by the anomalous flow^23^; similarly, they can lead to extreme large snow accumulations followed by extensive flood events during the melting season^24^. The simulated increase in the blocking event frequency throughout the 16th–18th centuries agrees with the results of reconstructions of SLP and atmospheric circulation during this period^25–27^. We thus conclude that such persistent anomalous conditions are largely responsible for the winter amplification of the LIA cooling in northern and central Europe.

In contrast to winter, the simulated upper-ocean cooling (Fig. 2b) and sea ice growth during LIA summers lead to negligible changes in the ocean surface heat flux in the North Atlantic/Arctic region (Fig. 3a). The ISR-OHT reduction is associated with minor, mostly non-significant (p > 0.05) changes in SLP and near-surface winds over Europe (Fig. 3b,c) and, thus, plays a minor role in modulating European summer temperature variability on decadal and longer time scales (Suppl. Figure 4). Therefore, we conclude that the proposed mechanism for the winter amplification of the European LIA cooling is inactive in summer.

We next discuss the relative role of the AMOC, NAO, and SPG (indices in Suppl. Figure 6a) in driving the simulated ISR-OHT reduction associated with the European LIA cooling. It is important here to stress that separating the AMOC and the SPG is fundamental to capturing core processes driving climate variability in the North Atlantic/Arctic region, because the AMOC and the SPG dominate the oceanic heat transport at different latitudes^28^. Both meridional and latitudinal changes in the Atlantic Ocean flow need to be analyzed together^29^.

AMOC anomalies are mostly non-significant (p > 0.05) during the LIA, and there is no robust indication in the ensemble of an AMOC strengthening or weakening in these centuries (Suppl. Figure 6). Furthermore, wavelet coherence analysis shows that centennial ISR-OHT changes leads those in the AMOC (Suppl. Figure 7). The winter amplification of the European LIA cooling is therefore not directly connected to AMOC variability.

Coherency analysis focused on the role of the NAO excludes that centennial/multicentennial ISR-OHT changes during the LIA are directly related to NAO variations (Suppl. Figure 7). In addition, none of the ensemble realizations present a phase shift in the winter NAO that explains the ISR-OHT reduction (Suppl. Figure 6). The most recent reconstructions of the winter NAO^19^ shows a series of century-long periods in the 18th to early 19th centuries, during which its mean phase remains statistically significantly (p < 0.1) negative (see Suppl. Figure 6). A similar analysis in our ensemble reveals that the European LIA cooling is not systematically associated with such century-long periods of anomalous NAO phase, which indeed can occur at almost any time in the past millennium (Suppl. Figure 6). For a decadally smoothed NAO index, none of the simulations correlate significantly (p < 0.05) with the reconstruction or with any of the other realizations (not shown). These results suggest that the simulated NAO variability on decadal and longer timescales arises mostly from internal climate dynamics, and therefore imply that we should not expect model–data agreement.

We also exclude that the winter SLP anomalies in Fig. 3b reflect a negative winter NAO pattern, because the two patterns are spatially uncorrelated during the LIA (Suppl. Figure 8). Therefore, our results do not support previous studies that link the European LIA cooling with a weakened northward heat transport by the AMOC^15, 16^ or with a persistent negative NAO^17^.

Rather, we find strong coherency between the simulated ISR-OHT and the SPG variability on centennial and multicentennial time scales in both winter and summer, with the two series in co-phase or with the SPG leading (Suppl. Figure 7). This result indicates that both the SPG and ISR-OHT variability are closely connected on these time scales and supports the idea that the long-lasting ISR-OHT reduction and, by extension, the winter amplification of the European LIA cooling are linked to a multicentennial SPG weakening. This weakening is consistently simulated in all ensemble realizations (Suppl. Figure 6). Further evidence of the leading role of the SPG results from the fact that only a SPG weakening, and not an AMOC weakening or a negative NAO phase, can explain the simulated anomalous pattern in near-surface temperatures that characterizes the entire North Atlantic/Arctic and European regions during these centuries in winter and summer (compare panels in Suppl. Figure 9).

A multicentennial weak SPG is self-sustained by its own dynamics through a redistribution of upper-ocean salinity (Suppl. Figure 10) and heat (indirectly shown via sea-surface temperatures in Fig. 2b) within the subpolar North Atlantic and Nordic Seas^30^. This redistribution is driven by a reduction in the SPG’s salt and heat transports to the Labrador and Nordic seas. The resultant anomalous upper-ocean freshening/increase in salinity in the western/eastern subpolar North Atlantic weakens the zonal gradient in seawater density that sets the SPG strength^30^. The surface freshening in the Labrador Sea is further sustained by an increase in the sea ice transport across the Denmark Strait due to colder upper-ocean conditions to the north^30^. No reduction in the wind stress curl driving the SPG is found during these centuries (not shown). Wavelet coherency analysis does not indicate that the weak SPG is driven by either AMOC or NAO variability (Suppl. Figure 11).

The simulated weakening of the SPG’s heat transport induces changes in the upper-ocean temperature and sea ice concentration that are compatible with reconstructed estimates during the LIA, including the cooling–warming dipole in the subpolar North Atlantic (dots in Figs 2b and 3a). The simulated colder, hence denser (not shown), ocean surface conditions might also explain the increase in δ^18^O (suggested as proxy for ocean surface density) that is measured during the LIA off North Iceland^31^: in particular between the periods 1575–1724 CE and 950–1250 CE (as in Fig. 2b), the increase amounts to 0.13‰, and is statistically significant (p < 0.05, using a two-tailed Student’s t test). Our findings also agree with oceanographic reconstructions of a weak SPG^32, 33^, a decrease in the Atlantic water’s influence on the North Iceland Shelf^34^, and a reduction in the northward heat transport to the Arctic in the Nordic Seas^35, 36^ during the LIA. Thus, reconstructed changes in this region, previously attributed to variations in the “surface AMOC”, would mostly be capturing changes in the gyre circulation, with no clear contributions from changes in the AMOC^18^ or NAO^19^. We conclude that the winter amplification of the European LIA cooling is directly related to widespread North Atlantic/Arctic cooling driven by a weak SPG.

The results presented here differ from a previous explanation that attributes weak SPG episodes over the past millennium to atmospheric blocked conditions during periods of low solar activity^37^. In contrast, our simulations indicate that a weak SPG induces such atmospheric conditions, with the onset of the SPG weakening coinciding with a solar maximum in the late 16th to early 17th centuries^30^. Other works highlight the role of strong volcanic forcing during the 13th–14th centuries in the onset of the LIA cooling^15^; yet, this period is well before the coldest phase of the European LIA (in the c. 16th–18th centuries) considered here. Nonetheless, our earlier work on the SPG weakening during the LIA (ref. 30) and the results presented here are consistent with recent studies that attribute the LIA cooling mainly to increased volcanic activity in the late 16th to 18th centuries, with minor contributions from other external forcings^13, 14, 20^.

As a study based on a single climate model, it indeed may be influenced by the biases the model presents^38^. In regard to this study, relevant biases include, for example, the erroneous path of the Gulf Stream and a cold bias in the subpolar North Atlantic, which could potentially influence decadal climate variability of the region^39^. Another possible source of uncertainty is the employed reconstructions of the external forcings defined within the CMIP5 protocol^40^. However, even though these forcings have recently been updated^41^, they do not substantially differ from those used here^41^. Although it is unclear whether and how these issues have biased our results, we feel confident in the conclusions presented here, in view of the good agreement that is consistently found between our model results and the variety of considered climate reconstructions shown, for example, in Figs 1, 2 and 3.

In summary, based on analysis of state-of-the-art climate simulations and reconstructions, we provide a novel and coherent picture of the dynamical mechanism that explains the strong winter amplification, compared to summer, of the European LIA cooling, and in which the connection between the SPG and the European climate is the key to disentangling the contrast between these seasons. Our analysis clearly illustrates that long-lasting episodes of regional winter cooling in northern and central Europe can be explained by variations in the SPG strength and related oceanic heat transport, through changes in the Arctic sea ice extent and subsequent ocean–atmosphere interactions. Ongoing and future changes in the SPG strength, associated with AMOC slowdown^28^, under anthropogenic global warming could therefore have important implications for the European and Arctic climates because they could contribute to enhancing sea ice retreat^28, 35^. The dominant role of the SPG in shaping multicentennial climate variability in the North Atlantic/European sector demonstrated here calls for improved understanding of coupled dynamics in the subpolar Atlantic, and encourages to surpass common paradigms, such as the AMOC or NAO, when characterizing its variability.

## Methods

### Max Planck Institute Earth System Model (MPI-ESM)

The analyzed simulations were produced with the MPI-ESM for paleo-application (MPI-ESM-P). This version includes the atmosphere general circulation model ECHAM6^42^ at T63 horizontal resolution (1.875°) and 47 vertical levels that resolve the stratosphere up to 0.01 hPa. The ocean/sea ice model MPIOM^38^ has a conformal mapping horizontal grid of nominal 1.5° resolution that converges toward the North Pole, situated over Greenland, and that increases the resolution in the North Atlantic up to 15 km, with 40 unevenly spaced vertical levels.

### MPI-ESM simulations

We use an ensemble of three full-forcing transient climate simulations of the past millennium (Past1000-R1, R2, and R3; between 850 CE and 2005 CE), following the protocol of the Paleoclimate Modeling Intercomparison Project, Phase 3 (PMIP3) within the Coupled Model Intercomparison Project, Phase 5 (CMIP5)^40, 43^. The prescribed external forcing included volcanic aerosol properties^44^; total solar irradiance reconstructed for the periods 850–1849 CE and 1850–2005 CE [refs 45, 46 respectively], with an artificial 11-year cycle imposed over the pre-industrial period; atmospheric concentrations of the most important well-mixed greenhouse gases and anthropogenic aerosols^40^; changes in global-cover and agricultural areas^47^; and annual changes in the Earth’s orbital parameters^40^. Additionally, we use an 1156-year control simulation under constant preindustrial (1850 CE) boundary conditions (PiControl) as a reference for the forced runs. For a more detailed description of the experimental setup and forcings used, refer to ref. 28.

### Indices

The main indices that are used here are defined as:

#### Iceland–Scotland Ridge oceanic heat transport (ISR-OHT)

The ISR-OHT is calculated as the volume transport through the Iceland–Scotland Ridge (indicated in Suppl. Figure 3b) from the bottom to the surface, multiplied by the temperature, a reference density equal to 1025 kgm^−3^, and the specific heat of seawater, equal to 4000 Jkg^−1^K^−1^, and using a reference temperature equal to 0 °C.

#### Subpolar gyre strength

The vertical structure of the mean currents in the subpolar North Atlantic allows describing the circulation of the SPG using the barotropic streamfunction, itself derived from the vertically integrated currents. We define an index for the SPG strength as the absolute value of the barotropic streamfunction spatially averaged between 50°N–65°N and 10°W–60°W.

#### Meridional overturning index

We characterize the variability of the AMOC strength via the meridional overturning index (MOI; ref. 48). This is defined as the average of the zonally integrated overturning streamfunction in the North Atlantic between 35°N and 45°N at 1000 m depth, i.e., where the climatological maximum of the simulated AMOC is located in PiControl (see, for example, Suppl. Figure 6b).

#### North Atlantic Oscillation

The NAO is defined as the first Principal Component of the SLP anomalies (with respect to the average of the preindustrial period, 850–1849 CE) in the North Atlantic sector, between 20°N–90°N and 80°W–40°E. The NAO is calculated in both winter (DJF) and summer (JJA), when it explains about 55% and 45% of the total variance respectively. The patterns are shown in Suppl. Figure 8.

### Statistical Significance

Statistical significance of anomalies in panels of Fig. 1b is calculated based on a two-tailed Student’s *t* test, in which effective degrees of freedoms and serial autocorrelation are taken into account^49^. In Figs 2 and 3, statistical significance is calculated based on the likelihood of a random occurrence of the signal in PiControl: signals detected in the forced runs are compared to analogs obtained by randomly sampling them 500 times across the full length of PiControl; the percentile 5th and 95th of the empirical anomaly distribution are subsequently used to determined confidence levels.

### Reconstruction dataset

We use the reconstructed fields of temperature variability of Europe over the Common Era in summer and winter from ref. 2 (LUT16) and 3 (LUT04), respectively. The winter reconstruction extends back until 1500 CE; additionally, the magnitude of its reconstructed LIA cooling appears underestimated with respect to the simulation in both seasons (Suppl. Figure 2). This is a consequence of a well-known loss of low-frequency variability by the methodology employed (see, for example, Supplementary Material in ref. 2, and ref. 21). This deficiency has already been addressed in the new reconstructed temperature field in summer in ref. 2, for which the magnitude of the LIA cooling is, in fact, comparable to the one simulated (Fig. 1b).

Despite the above-mentioned deficiency in the temperature reconstruction in ref. 3, the coldest winter decade (1689–1698 CE) exhibits anomalies comparable to those simulated (Suppl. Figure 12). Interestingly, this decade is also one of the coldest in the simulation, which corresponds with the so-called “climax of the LIA”^50^ during the Maunder Minimum of solar activity (see, for instance, ref. 51). However, the second and third coldest decades in the winter reconstruction show anomalies of smaller amplitude (Suppl. Figure 12), which might explain the overall underestimation of the LIA cooling in the reconstruction (Fig. 1b); nevertheless, the coldest anomalies are found mostly in northern and central Europe in all cases.

We further use independent winter temperature reconstructions to support the magnitude and spatial pattern of the simulated winter amplification of the LIA cooling in northern and central Europe (dots in Fig. 1b). These reconstructions are for Stockholm (Sweden, 59.3°N–18.0°E; ref. 9), Tallinn (Estonia, 59.3°N–24.7°E; ref. 10), northern (69.0°N–22.0°E) and southern Norway (61.5°N–8.0°E; the data were obtained by digitalizing Figure 4 in ref. 52; anomalies are with respect to 1950 CE), and central Europe (49.0°N–13.0°E, at the center of the region studied in ref. 7].

References of the reconstructions of SST (Fig. 2b) and sea ice conditions (Fig. 3a) are given in Suppl. Figure 3. Changes during the LIA are calculated with respect to the Medieval Climate Anomaly (c. 950–1250 CE) as some records do not cover the 20th century. Additionally, face colors in the plotted circles in Figs 2b and 3a are not adapted to represent exact anomaly in SST or sea ice concentration during the LIA (in contrast to those in Fig. 1b) because some of the records are only proxies for SST or sea ice conditions rather than SST or sea ice concentration itself (e.g., δ^18^O for SST, and IP25 or relative changes in certain foraminiferal species for sea ice conditions). Therefore, the color scale is simplified to indicate only whether sites show anomalous cooling and expanded sea ice (blue) or warming (red) during the LIA.

### Data Availability

Primary data and scripts used in the analysis and other supplementary information that may be useful in reproducing the author’s work are archived by the Max Planck Institute for Meteorology, and can be obtained by contacting publications(at)mpimet.mpg.de. All Figures were produced using Pyferret v. 7.0., which is freely provided by NOAA. (Information is available at http://ferret.pmel.noaa.gov/Ferret/).

## Electronic supplementary material

Supplementary Material
