# Supplementary material for: Winter amplification of the European Little Ice Age cooling by the subpolar gyre
Source: Sci Rep. 2017 Aug 30;7:9981. doi: 10.1038/s41598-017-07969-0 (PMC5577174; doi:10.1038/s41598-017-07969-0)
Supplement: Supplementary file 1 — Supplementary Material [file 41598_2017_7969_MOESM1_ESM.pdf]

Winter amplification of the European Little Ice Age cooling by the subpolar gyre: Supplementary Material

Eduardo Moreno-Chamarro[1,2,3,*], Davide Zanchettin[4], Katja Lohmann[1], Jürg Luterbacher[5,6], and Johann H. Jungclaus[1]

1. Max Planck Institute for Meteorology, Hamburg, Germany
2. International Max Planck Research School on Earth System Modeling, Hamburg, Germany
3. Now at Department of Earth, Atmospheric and Planetary Sciences, Massachusetts Institute of Technology, Cambridge, USA
4. University Ca'Foscari of Venice, Department of Environmental Sciences, Informatics and Statistics, Mestre, Italy
5. Department of Geography, Justus Liebig University of Giessen, Giessen, Germany
6. Center of International Development and Environmental Research, Justus Liebig University of Giessen, Giessen, Germany
* Corresponding author: chamarro(at)mit.edu

a. Summer

	LUT16	R1	R2	R3	
**LUT16**		0.65	0.75	0.62	1500-2003
**R1**	0.43		0.75	0.76	
**R2**	0.49	0.61		0.69	
**R3**	0.44	0.59	0.58		
		850-2003			

b. Winter

	LUT04	R1	R2	R3	
**LUT04**		0.35*	0.38*	0.10*	1500-2003
**R1**	-		0.47	0.46	
**R2**	-	0.39		0.20*	
**R3**	-	0.29	0.16*		
		850-2003			

Suppl. Table 1: Correlation coefficients between the reconstructed and the simulated European temperatures shown in Figure 1a in summer (June–August, JJA; **a**) and winter (December–February, DJF; **b**) for the periods 850–2003 CE and 1500–2003 CE (yellow/gray panels below/above diagonal respectively). Asterisks denote statistically non-significant values ($p > 0.05$), accounting for effective degrees of freedom and autocorrelation in time series [Von Storch and Zwiers, 2001]. Table produced in *LibreOffice Calc v. 5.1.4.2*

.

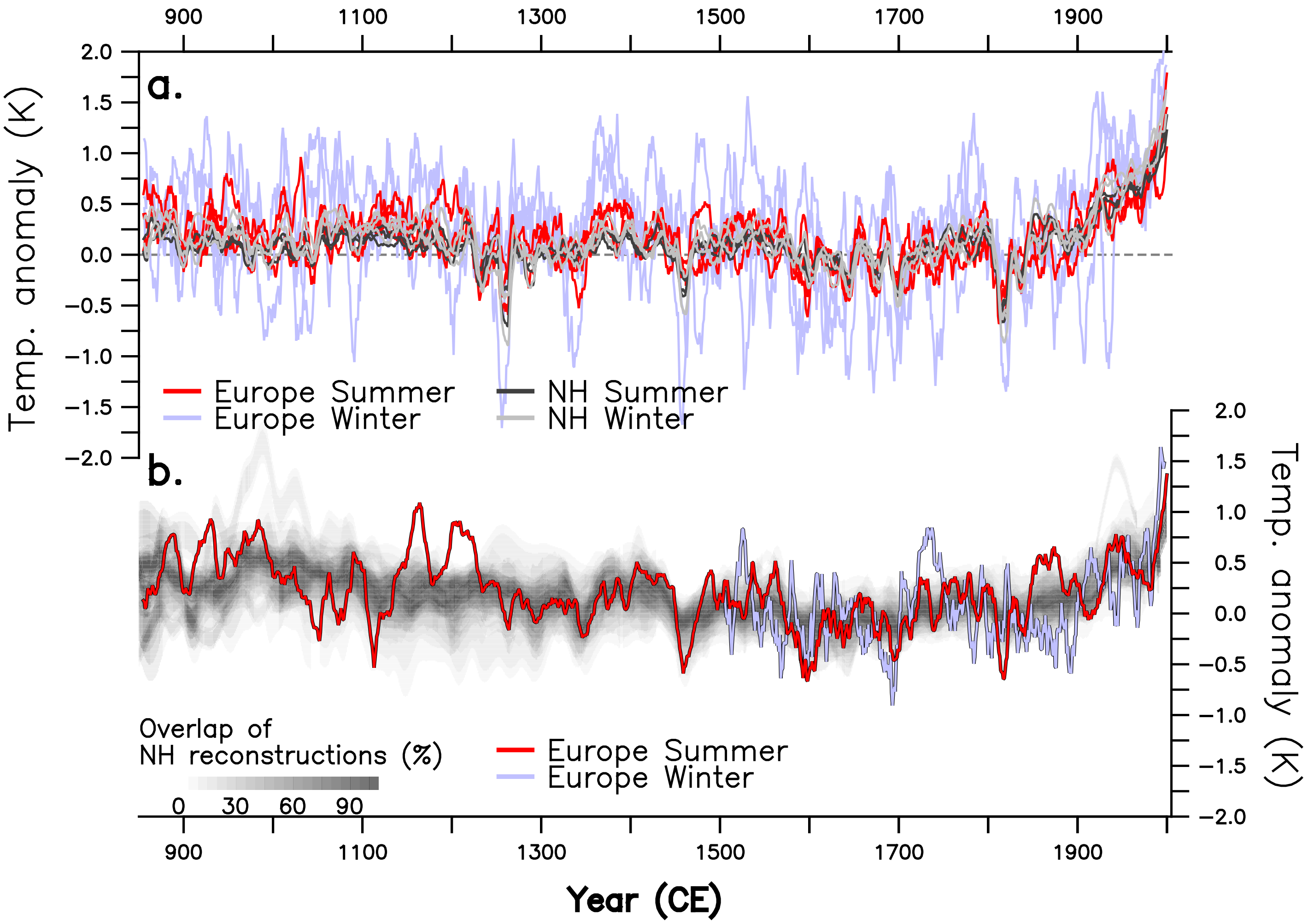

Suppl. Figure 1: European temperature anomaly (in K) in winter (DJF; light blue) and summer (JJA; red), simulated in the Past1000 ensemble **(a)** and reconstructed **(b)**, as in Figure 1a. Also in **(a)**, simulated Northern Hemisphere (NH) mean temperature anomaly (in K) in summer (dark gray) and winter (light gray), smoothed with an 11-year running mean. Also in **(b)**, reconstructed Northern Hemisphere mean temperature anomaly (in K), with gray shading illustrating the overlap of available NH temperature reconstructions (in percent), from Masson-Delmotte et al. [2013]. All anomalies are calculated with respect to 1500–1850 CE.

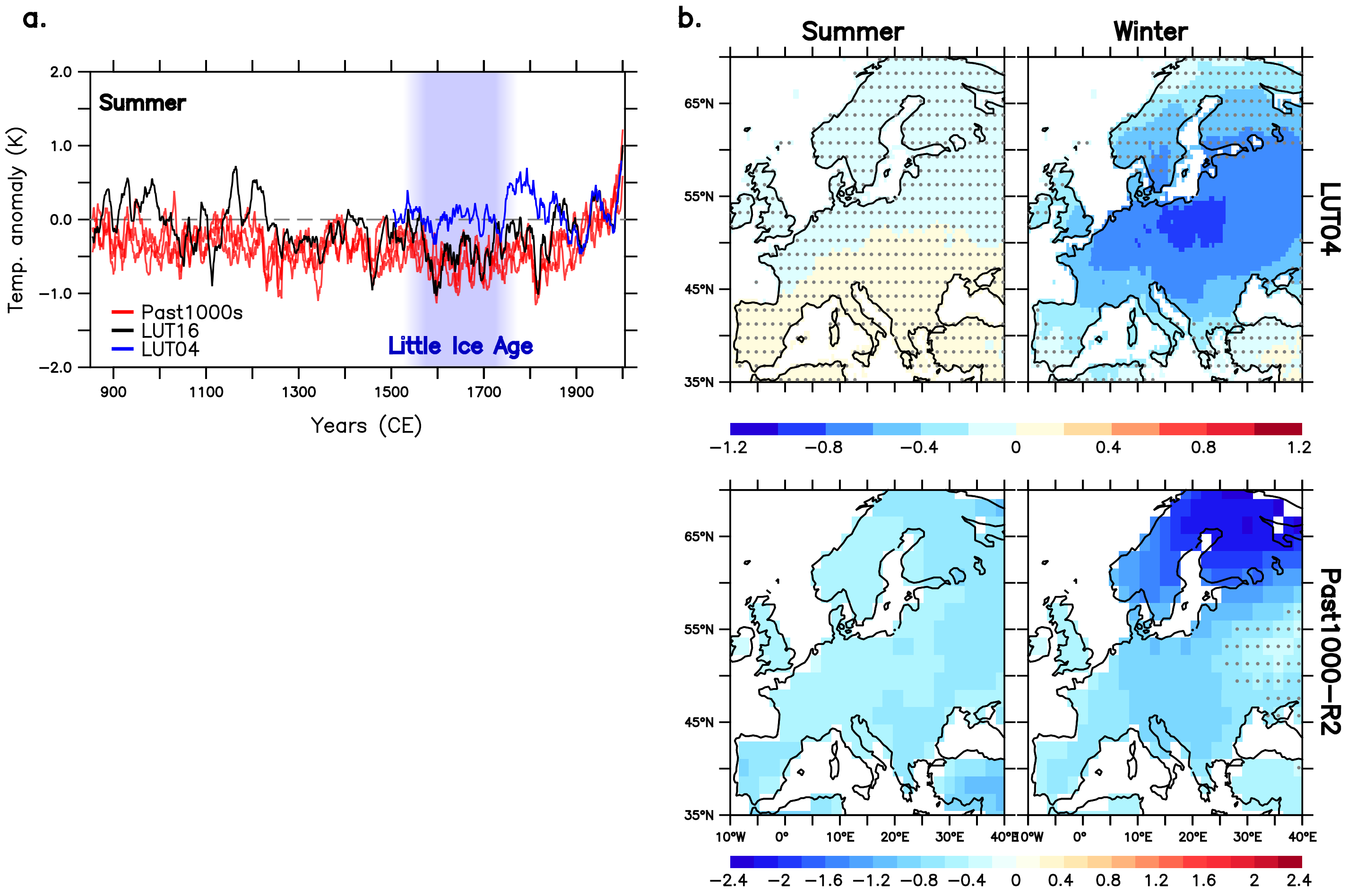

Suppl. Figure 2: **a,** European summer (JJA) temperature anomaly (in K), as in Figure 1a, simulated in the Past1000 ensemble (red), and reconstructed in Luterbacher et al. [2004] (LUT04; blue) and Luterbacher et al. [2016] (LUT16; black). **b,** Land temperature anomalies (in K) for the period 1575–1724 CE (shading in **a**) with respect to 1901–1990 CE, simulated in Past1000-R2 (bottom) and reconstructed in LUT04 (top), in summer (left) and winter (right). Note that, in contrast to Figure 1b, color scales for the simulated and the reconstructed anomalies are different; this allows highlighting the systematic low-frequency-variability underestimation that the LUT04's reconstruction presents. Stippling masks statistically non-significant anomalies at the 5 % level. Maps were generated in *Pyferret v. 7.0.* (Information is available at http://ferret.pmel.noaa.gov/Ferret/).

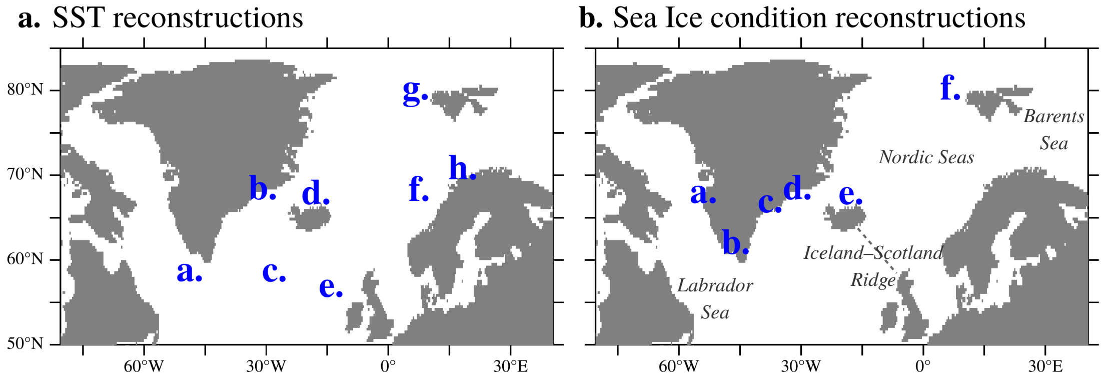

Suppl. Figure 3: Core sites of the reconstructions of **(a)** sea-surface temperature shown in Figure 2c: **a**, Moffa-Sánchez et al. [2014], **b**, Miettinen et al. [2015], **c**, Miettinen et al. [2012], **d**, Sicre et al. [2008], **e**, Richter et al. [2009], **f**, Berner et al. [2011], **g**, Spielhagen et. al. [2011], and **h**, Hald et al. [2011]; and of **(b)** sea ice conditions shown in Figure 3a: **a**, Sha et al. [2016], **b**, Jensen et. al. [2004], **c**, Andresen et al. [2013], **d**, Miettinen et al. [2015], **e**, Massé et al. [2008], and **f**, Werner et al. [2011]. The main oceanographic components mentioned throughout the manuscript are shown in **(b)**. Maps were generated in *Pyferret v. 7.0.* (Information is available at http://ferret.pmel.noaa.gov/Ferret/).

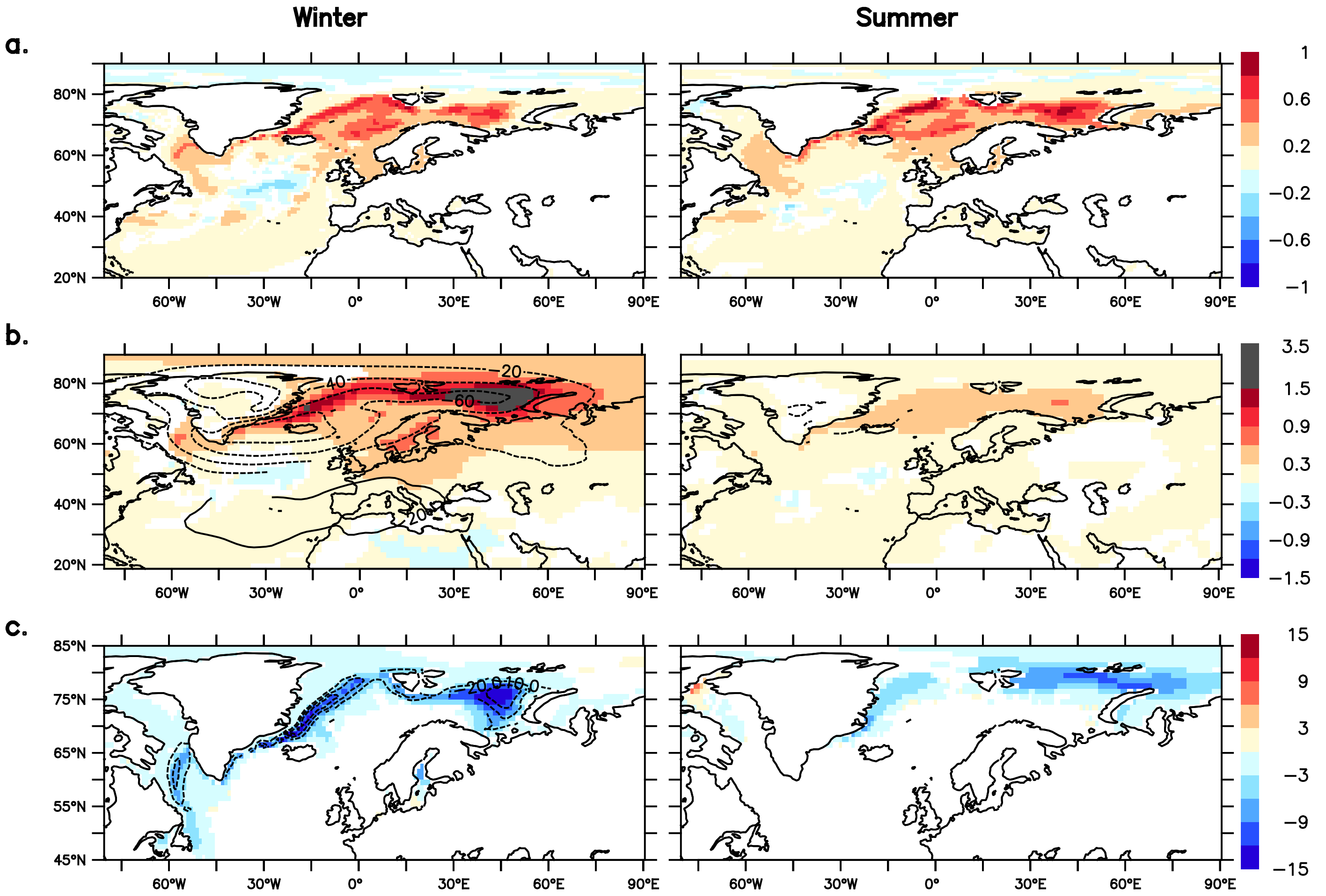

Suppl. Figure 4: In Past1000-R2, regression coefficients of **(a)** sea-surface temperature (in K), **(b)** near-surface (2 m) air temperature (SAT; shading, in K) and sea-level pressure (SLP; contours at 20-Pa intervals), and **(c)** sea ice concentration (shading, in percent of area) and ocean surface downward heat flux (contours at 10-W/m^2 intervals) onto the standardized Iceland–Scotland Ridge oceanic heat transport (ISR-OHT) in winter (DJF; left panels) and summer (JJA; right panels). Sea ice concentration is in late winter (March) and late summer (September), when it reaches its climatological maximum and minimum extension respectively. Note the same color scale for both panels in **a**–**c**, which is also adapted for a better view of the values over Europe in **b**. Only statistically significant values at the 5 % level are shown, based on two-tailed Student's t test in the PiControl climatology. Calculations are performed after applying an 11-year running mean and for the preindustrial period, 850–1849 CE, to avoid possible spurious effects due to anthropogenic influences. Maps were generated in *Pyferret v. 7.0.* (Information is available at http://ferret.pmel.noaa.gov/Ferret/).

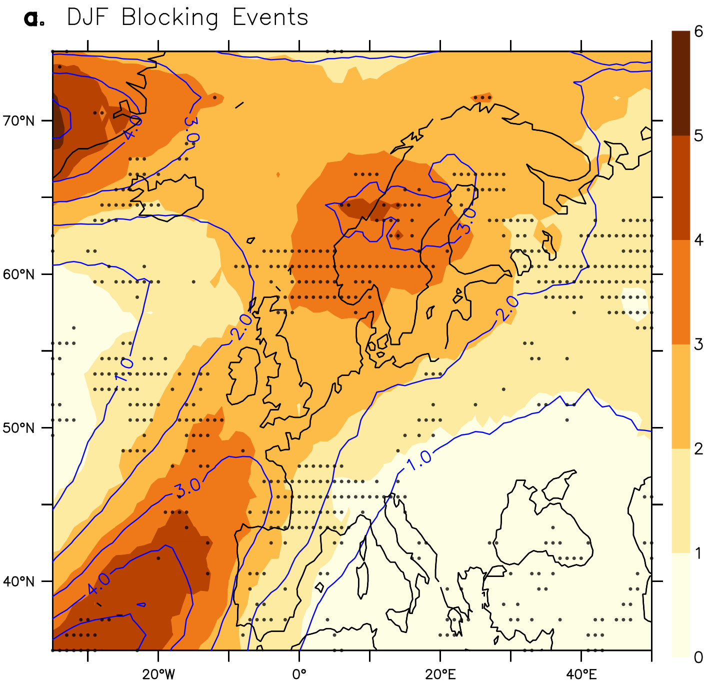

Suppl. Figure 5: a, Wintertime (DJF) blocking frequency for the period 1575–1724 CE (shading) in Past1000-R2, calculated using the indicator described in Scherrer et al. [2006]. Note that each increase of one unit in the blocking index corresponds to five or more additional days under blocked atmospheric situations. Contours represent the climatological mean in PiControl. Stippling masks statistically significant anomalies between the two at the 5 % level, based on the likelihood of occurrence of the signal in PiControl. Map was generated in *Pyferret v. 7.0.* (Information is available at http://ferret.pmel.noaa.gov/Ferret/).

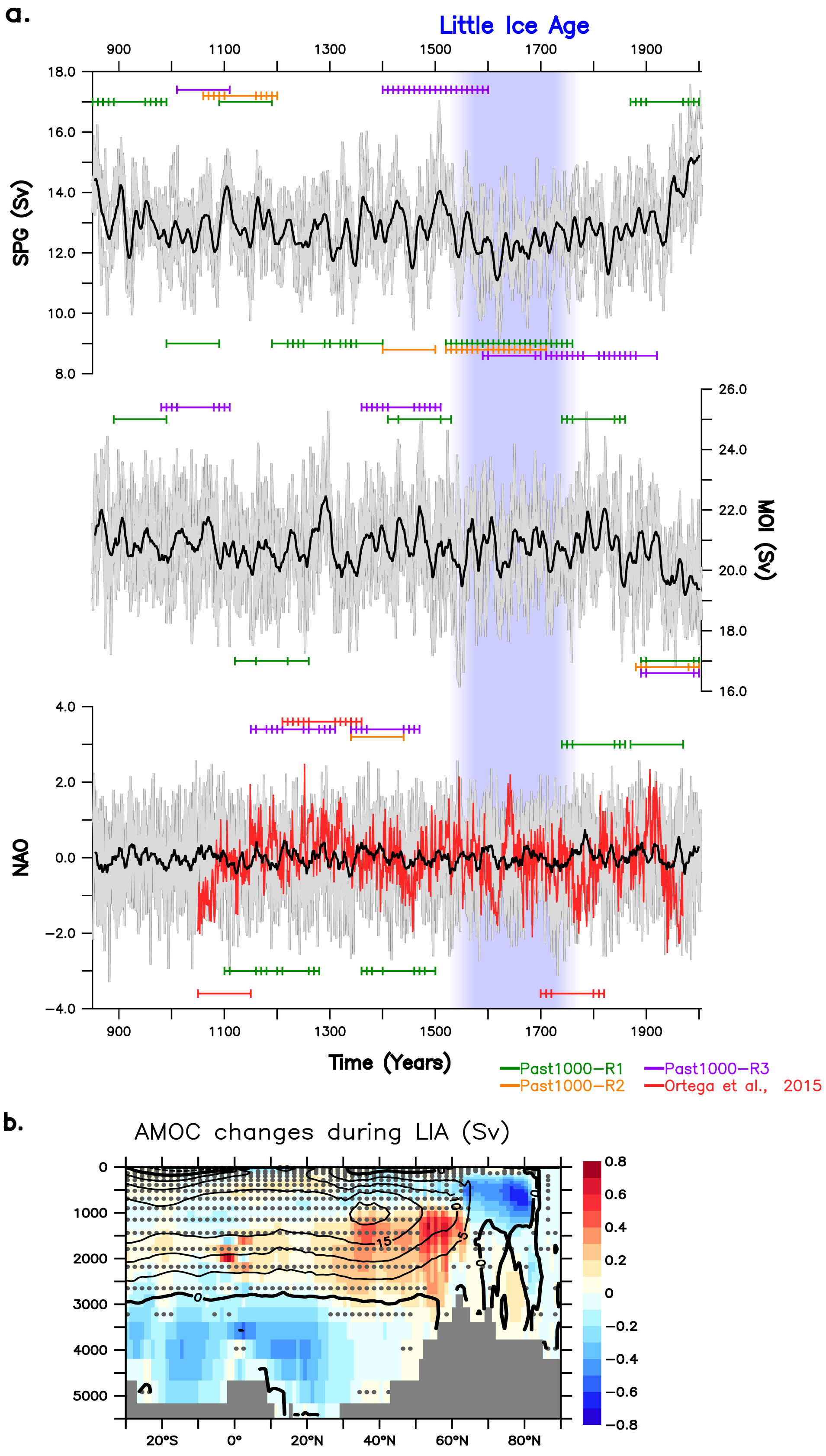

Suppl. Figure 6: (Caption next page.)

Suppl. Figure 6: (Previous page.) **a,** Annual mean subpolar gyre (SPG, in Sv; top) strength and meridional overturning index (MOI, in Sv; center), and wintertime (DJF) North Atlantic Oscillation (NAO; dimensionless; bottom). Indices for the SPG, MOI, and NAO are defined in the Methods section. Gray and black lines are respectively yearly values of the three Past1000 simulations and the 11-year running mean of the ensemble mean. Horizontal bars above (or below) the series enclose centuries (marked by tick marks) with positive (or negative) mean values that are significantly different ($p < 0.1$) from the PiControl climatological mean, or from zero for the reconstructed NAO (similarly as done in Ortega et al. [2015]). These periods are calculated, first, by correcting each Past1000's mean for the period 850–2005 CE by the long-term PiControl climatological average; then, and to assess whether a 100-year running average if different from this mean (or from zero), we apply a two-tailed Student's t-test, in which effective degrees of freedom and autocorrelation in time series are taken into account [Von Storch and Zwiers, 2001]. Since the latter imposes a more restrictive threshold than originally done in Ortega et al. [2015], we choose, instead, a confidence level of 10 %. **b,** Anomalies in the annual mean meridional overturning (in Sv; shading) for the period 1575–1724 CE (shading in **a**) in Past1000-R2 with respect to the PiControl climatology (shown in contours, in Sv). Stippling masks statistically non-significant anomalies at the 5 % level, based on the likelihood of occurrence of the signal in PiControl. Model bottom topography is shown in gray.

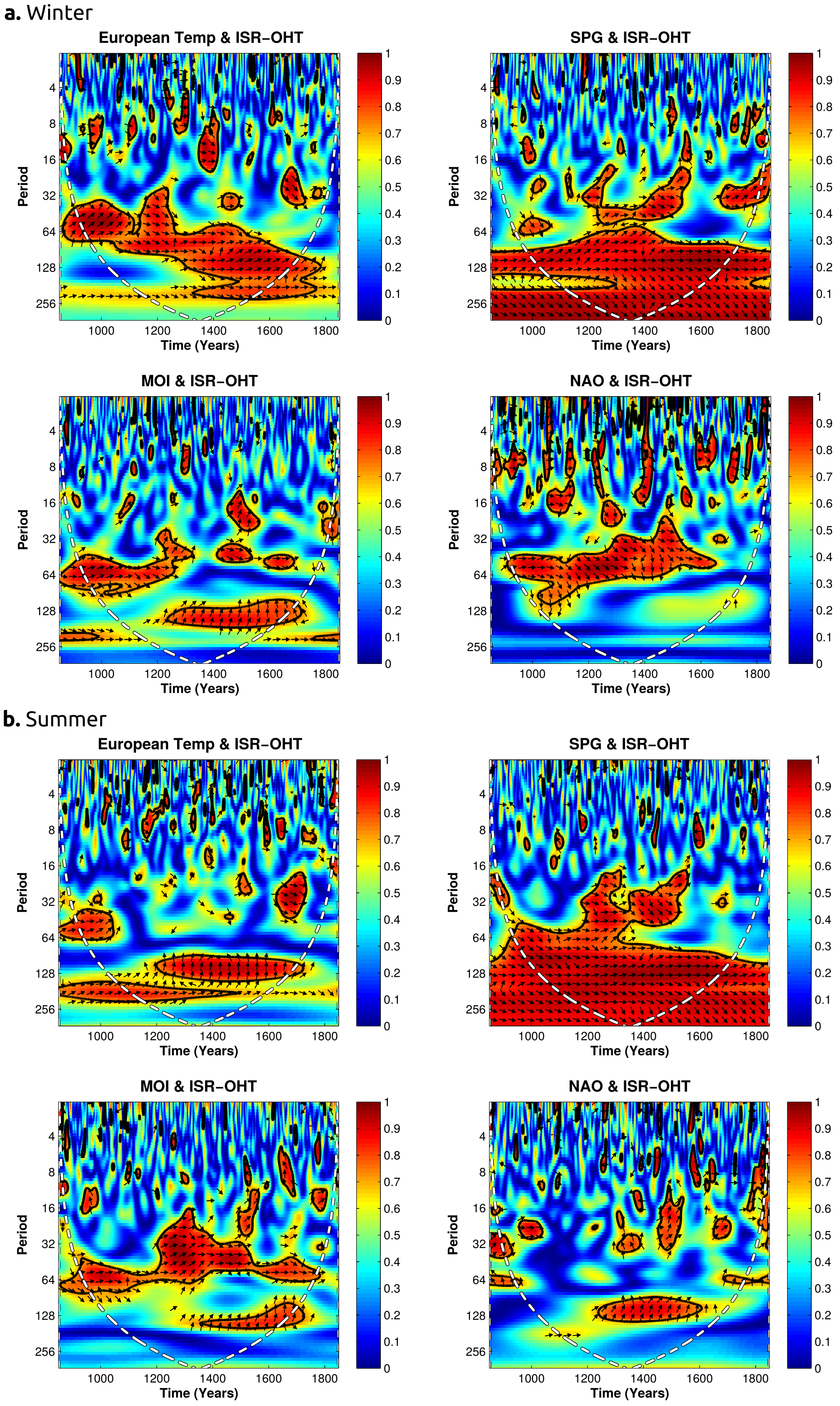

Suppl. Figure 7: (Caption next page.)

Suppl. Figure 7: (Previous page.) Wavelet coherency between the European temperature (from Figure 1a), SPG strength, MOI, and NAO (first time series; from Suppl. Figure 6a) and the ISR-OHT (second time series; from Figure 2a) in winter (DJF; **a**) and summer (JJA; **b**) for the preindustrial period, 850–1849 CE. Wavelet coherence analysis between two time series extracts regions in the time-frequency space with large common power and consistent phase relationship and thus suggests times when there might exist causality between processes underlying the two time series. Note, however, that significant coherency does not necessarily subtend causality between the two; this requires identification of a valid physical mechanism. Color shading indicates the strength of the coherence, with warm colors within black contours significant at the 5 % level against red noise. The direction of the arrows indicates the phase relationship between the two wavelet transform: right arrows indicate that the two series are in co-phase; left indicates that the two are in anti-phase (π phase angle); and down/up arrows indicate that the second series lags/leads the first one in quadrature (one fourth of the cycle at that period), if the two series are positively correlated. The cone of influence (white dashed line) defines the area in which the border effect does not influence the wavelet spectra.

We note that coherent AMOC and ISR-OHT variability is diagnosed on centennial timescales in winter for the period 1200–1700 CE; the phasing indicates that the ISR-OHT robustly leads the AMOC by roughly 30 years.

The NAO shows broadband coherency with the ISR-OHT on decadal to multidecadal time scales in winter, when the NAO tends to lead ISR-OHT changes, although with remarkably variable phasing. This multidecadal coherency, however, does not persist beyond 1600 CE, when we find the coldest centuries over Europe (Figure 1a). In summer, coherency between the NAO and the ISR-OHT occurs mostly on centennial time scales between c. 1250–1600 CE, with phasing indicating that the ISR-OHT leads the NAO by about 30 years. There is, however, overall less agreement in summer than in winter between the the ISR-OHT and the NAO.

Coherency between the SPG and the ISR-OHT is particularly strong on centennial and multicentennial time scales in both winter and summer, with both series in co-phase or with the SPG leading.

Figure produced in *Matlab R2013a*, using the algorithm described in Grinsted et al. [2004].

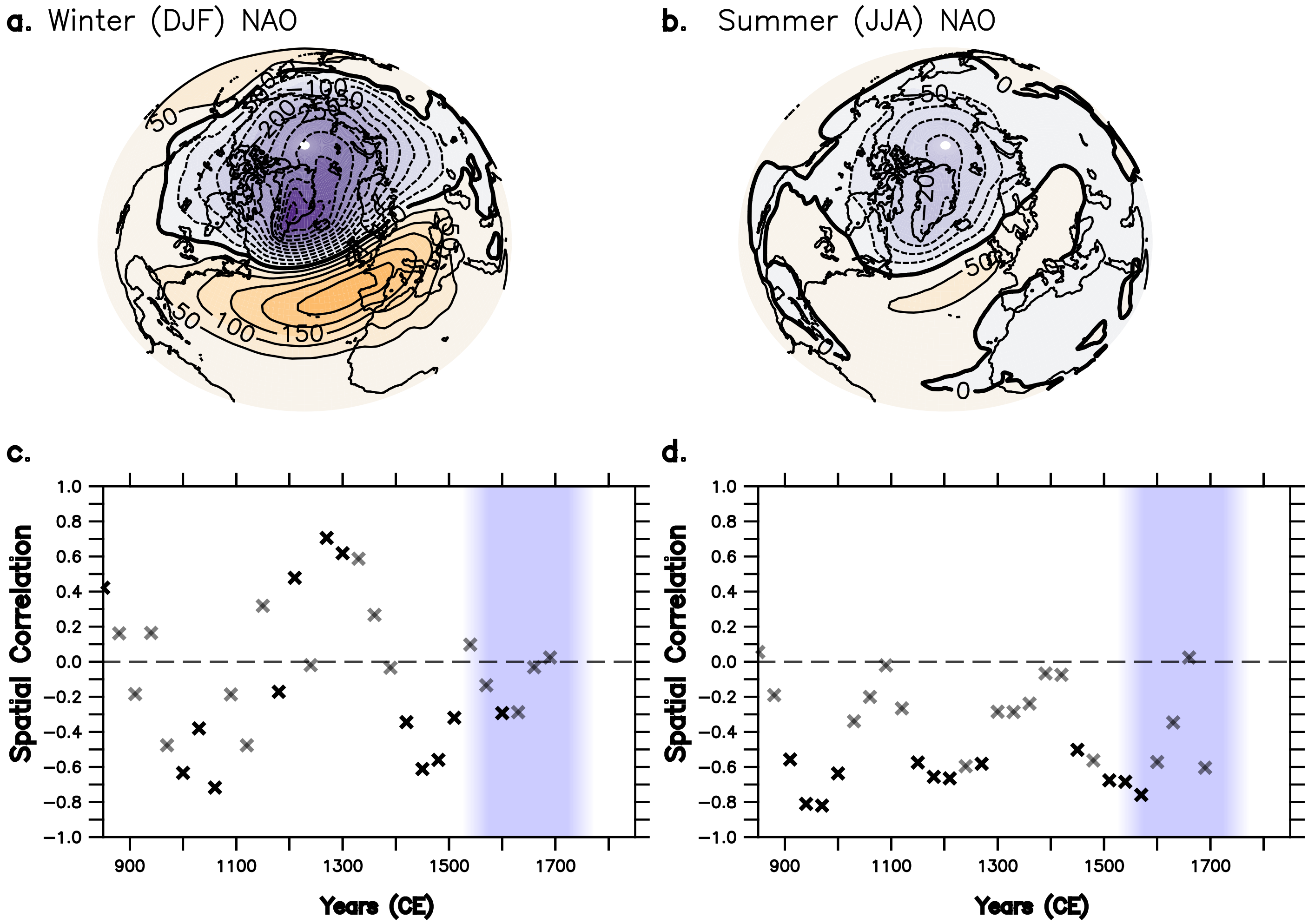

Suppl. Figure 8: a–b, NAO in winter (DJF; **a**) and summer (JJA; **b**) as simulated in Past1000-R2 (see Methods). The patterns shown here (in Pa) are obtained by regressing hemispheric SLP anomalies onto the time series of the leading principal component. **c–d**, Spatial correlation coefficients between a 150-year pattern of SLP anomalies calculated as in Figure 3b and the NAO in **(c)** winter and **(d)** summer between 80°W–40°E and 20°N–90°N, region where the NAO is calculated (see Methods section). Coefficients are calculated for the preindustrial period, 850–1849 CE, every 30 years, with significant values at the 5 % level accounting for effective degrees of freedom shown in bold. Values are plotted at the beginning of each period. Blue shading corresponds with the period 1575–1724 CE. Note that all correlation coefficients in summer appear to be biased toward negative values. This might be related to the fact that the difference between the Past1000 and the PiControl long-term climatologies in summer exhibits a negative NAO-like structure (not shown). Maps were generated in *Pyferret v. 7.0.* (Information is available at http://ferret.pmel.noaa.gov/Ferret/).

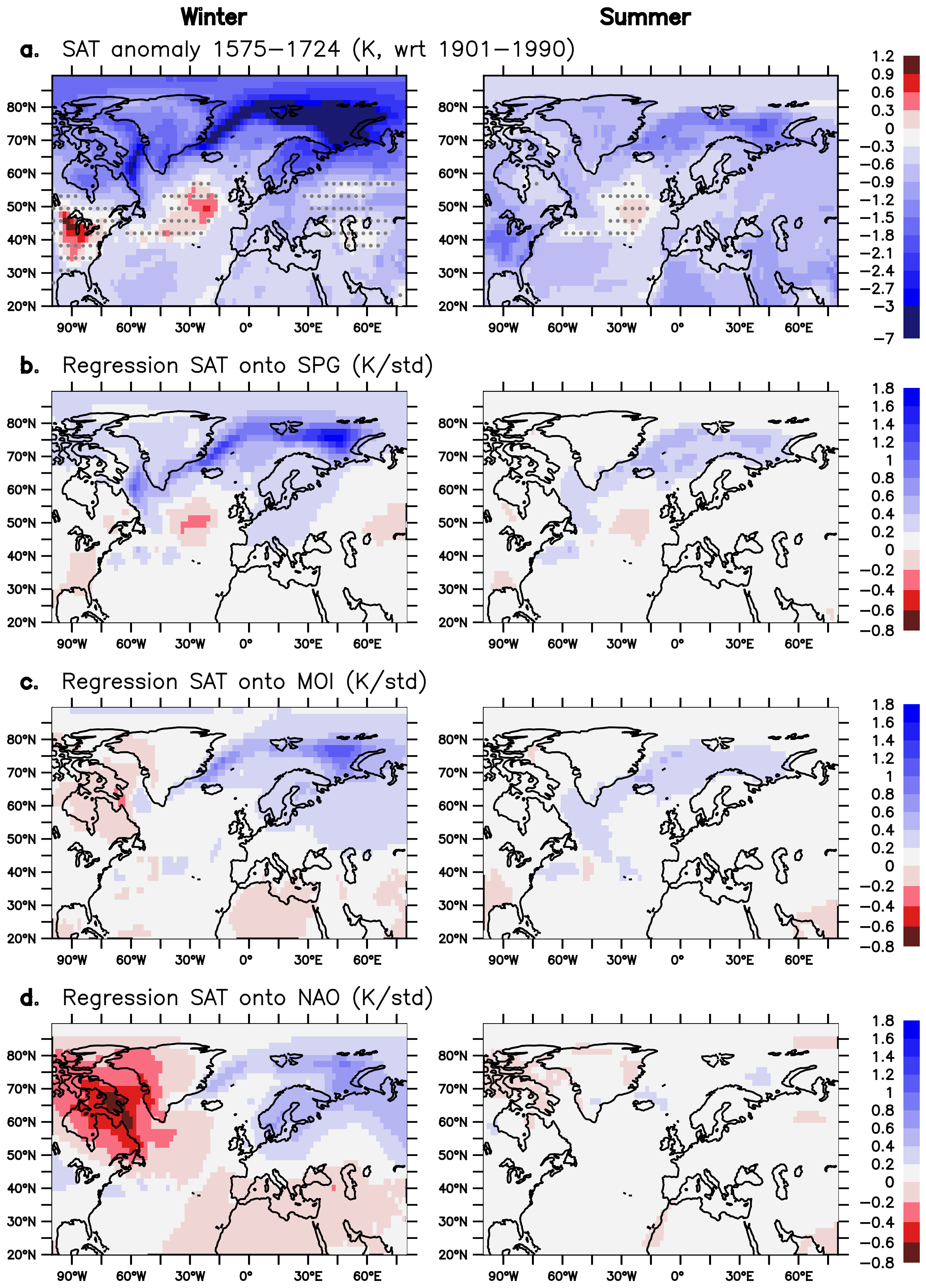

Suppl. Figure 9: (Caption next page.)

Suppl. Figure 9: (Previous page.) In Past1000-R2: **a,** SAT anomaly (in K) for the period 1575–1724 CE, with respect to 1901–1990 CE, in winter (DJF; left) and summer (JJA; right), as in Figure 1b but for the entire North Atlantic, Arctic, and European regions. Stippling masks statistically non-significant anomalies at the 5 % level. Note the same color scale for both seasons, which is also adapted for a better view of the values over both Europe and the subpolar North Atlantic. **b–d,** Regression coefficients of the SAT (in K per standard deviation, or std) onto the standardized indices for the SPG strength **(b)**, MOI **(c)**, and NAO **(d)** respectively, in winter (left) and summer (right). Calculations are performed after applying an 11-year running mean and for the preindustrial period, 850–1849 CE. Note the same color scale for all panels, which has additionally been reversed for a better comparison with panels in **a**. Maps were generated in *Pyferret v. 7.0.* (Information is available at http://ferret.pmel.noaa.gov/Ferret/).

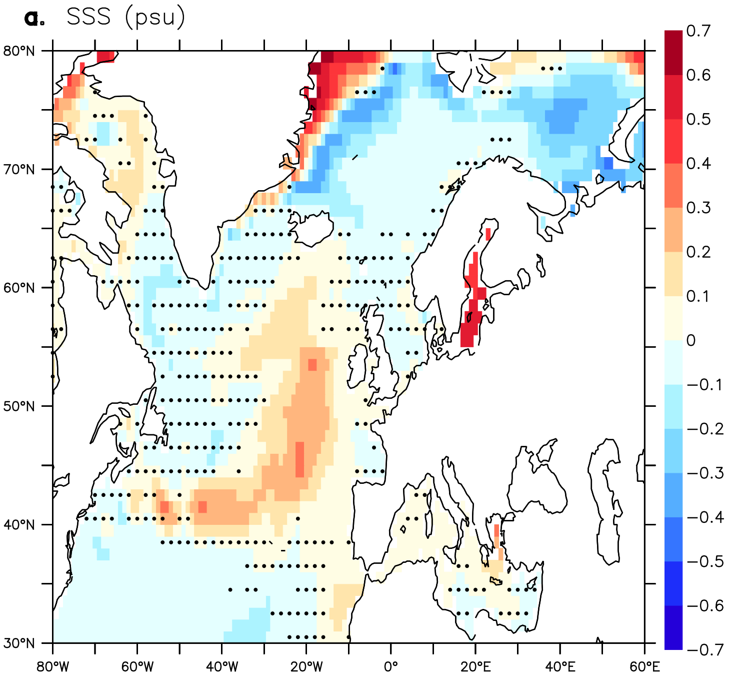

Suppl. Figure 10: a, In Past1000-R2, anomalies in annual mean sea-surface salinity (in psu) for the period 1575–1724 CE with respect to the PiControl climatology. Stippling masks statistically non-significant anomalies at the 5 % level, based on the likelihood of occurrence of the signal in PiControl. Map was generated in *Pyferret v. 7.0.* (Information is available at http://ferret.pmel.noaa.gov/Ferret/).

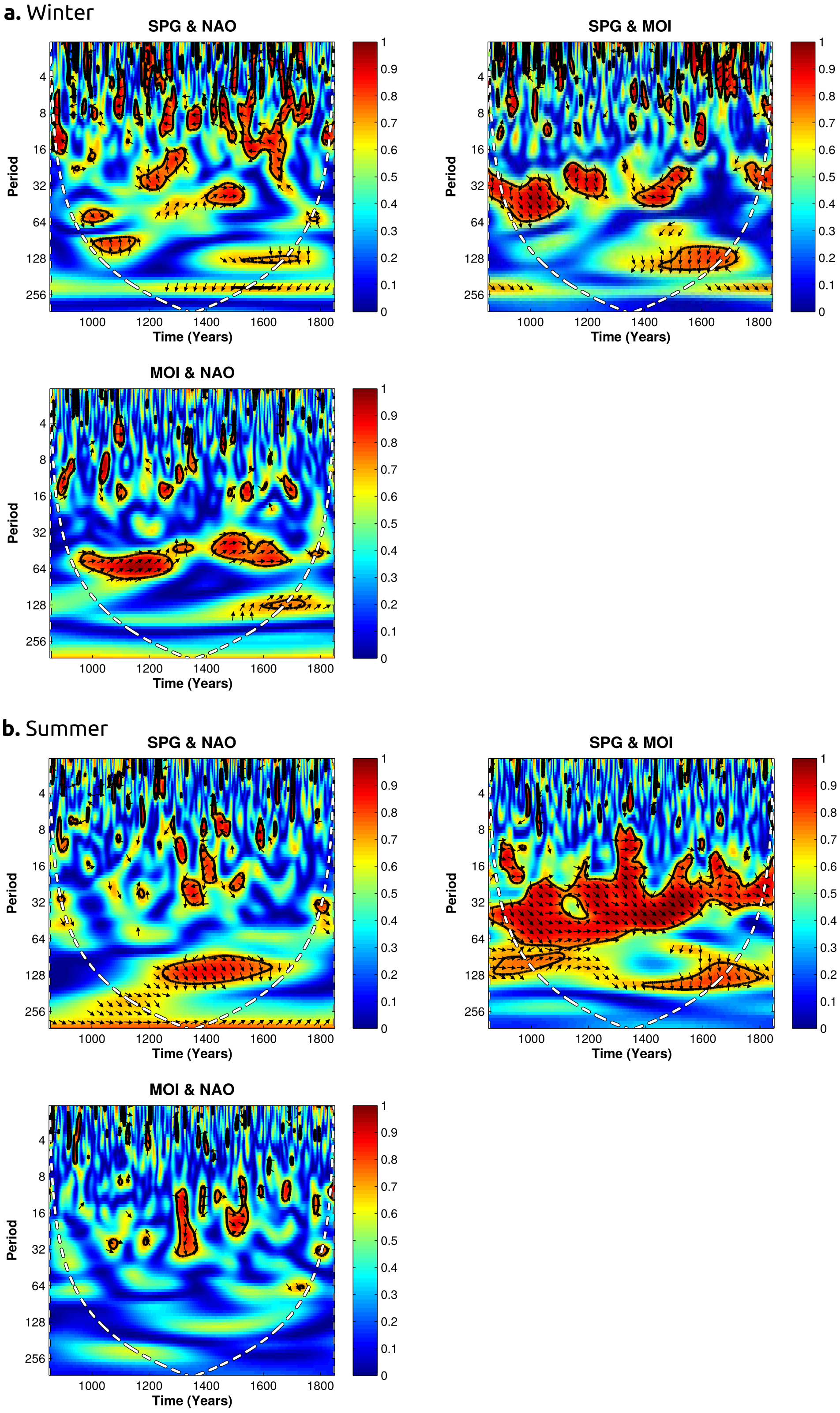

Suppl. Figure 11: Wavelet coherency (as in Suppl. Figure 7) between the SPG strength, MOI, and NAO in winter **(a)** and summer **(b)**. In each panel the first and the second time series are indicated.

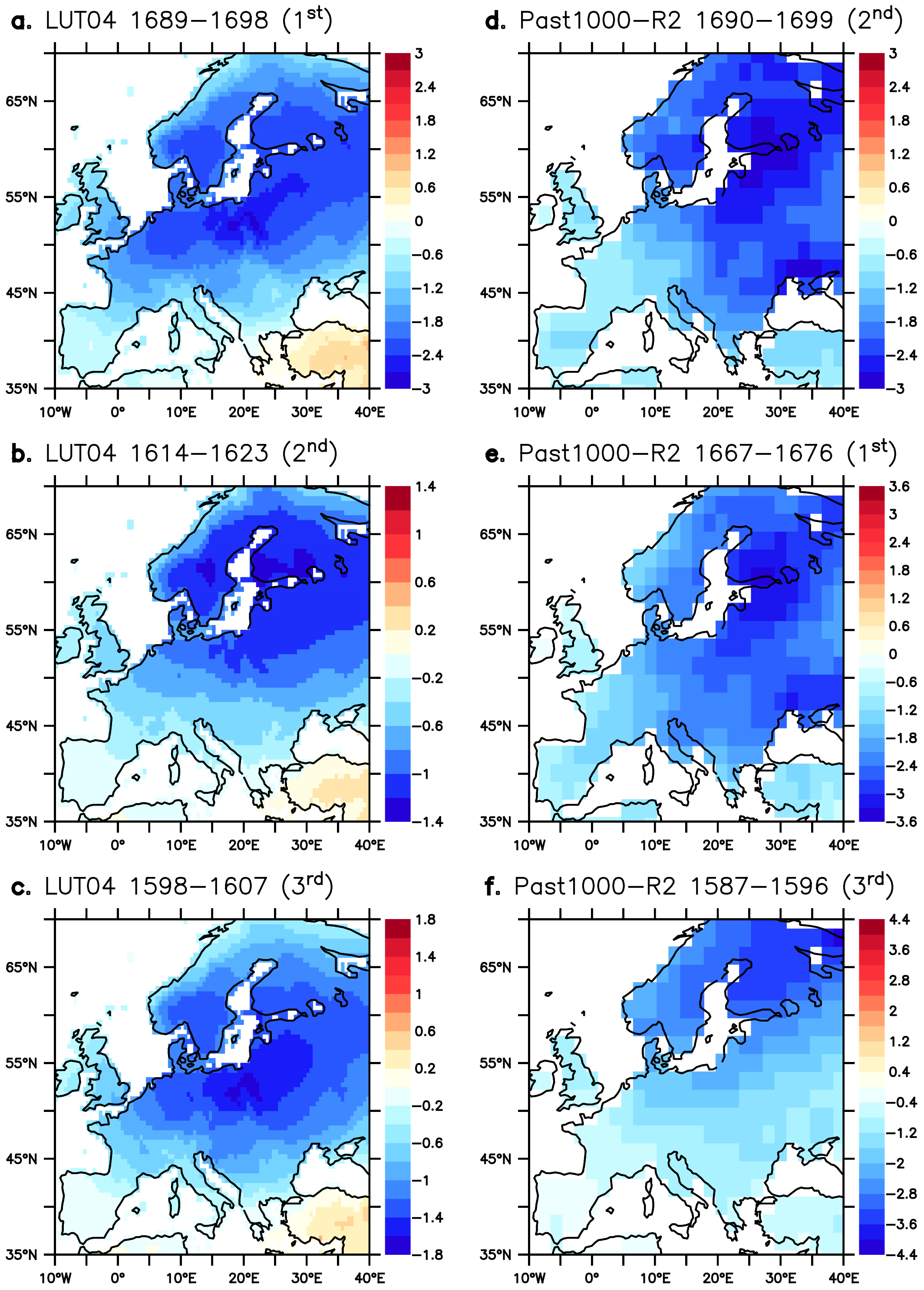

Suppl. Figure 12: Land temperature anomalies (in K) for the three coldest 10-year winter periods reconstructed in Luterbacher et al. [2004] (left) and simulated in Past1000-R2 (right). 1st indicates the coldest decade, 2nd the second coldest one, and so on. Note that the simulated 2nd decade is shown next to the reconstructed 1st one, since they cover the same period approximately. Maps were generated in *Pyferret v. 7.0.* (Information is available at http://ferret.pmel.noaa.gov/Ferret/).

References

Andresen, C., et al. Mid-to late-Holocene oceanographic variability on the Southeast Greenland shelf. *The Holocene*, **23(2),** 167–178 (2013).

Berner, K., Koç, N., Godtliebsen, F., and Divine, D. Holocene climate variability of the Norwegian Atlantic Current during high and low solar insolation forcing. *Paleoceanography*, **26(2)**, (2011).

Grinsted, A., Moore, J. C., and Jevrejeva, S. Application of the cross wavelet transform and wavelet coherence to geophysical time series. *Nonlinear Processes in Geophysics*, **11(5/6)**, 561–566 (2004).

Hald, M., Salomonsen, G. R., Husum, K., and Wilson, L. J. A 2000 year record of Atlantic Water temperature variability from the Malangen Fjord, northeastern North Atlantic. *The Holocene*, **21(7)**, 1049–1059 (2011).

Jensen, K. G., Kuijpers, A., Koç, N., and Heinemeier, J. Diatom evidence of hydrographic changes and ice conditions in Igaliku Fjord, South Greenland, during the past 1500 years. *The Holocene*,**14(2)**, 152–164 (2004).

Luterbacher, J., Dietrich, D., Xoplaki, E., Grosjean, M., and Wanner, H. European seasonal and annual temperature variability, trends, and extremes since 1500. *Science*, **303(5663)**, 1499–1503 (2004).

Luterbacher, J., et al. European summer temperatures since Roman times. *Environmental Research Letters*, **11(2)**, 024001 (2016).

Massé, G., et al. Abrupt climate changes for Iceland during the last millennium: evidence from high resolution sea ice reconstructions. *Earth and Planetary Science Letters*, **269(3)**, 565–569 (2008).

Masson-Delmotte, V., et al. Information from paleoclimate archives. *Climate Change*, 383–464 (2013).

Miettinen, A., Divine, D., Koç, N., Godtliebsen, F., and Hall, I. R. Multicentennial variability of the sea surface temperature gradient across the subpolar North Atlantic over the last 2.8 kyr,+. *Journal of Climate*, **25(12)**, 4205–4219 (2012).

Miettinen, A., Divine, D. V., Husum, K., Koç, N., and Jennings, A. Exceptional ocean surface conditions on the SE Greenland shelf during the Medieval Climate Anomaly. *Paleoceanography*, **30(12)**, 1657–1674 (2015).

Moffa-Sánchez, P., Hall, I. R., Barker, S., Thornalley, D. J., and Yashayaev, I. Surface changes in the eastern Labrador Sea around the onset of the Little Ice Age. *Paleoceanography*, **29(3)**, 160–175 (2014).

Ortega, P., et al. A model-tested North Atlantic Oscillation reconstruction for the past millennium. *Nature*, **523(7558)**, 71–74 (2015).

Richter, T. O., Peeters, F. J. C., and Van Weering, T. C. E. Late Holocene (0–2.4 ka BP) surface water temperature and salinity variability, Feni Drift, NE Atlantic Ocean. *Quaternary Science Reviews*, **28(19)**, 1941–1955 (2009).

Scherrer, S. C., Croci-Maspoli, M., Schwierz, C., and Appenzeller, C. Two-dimensional indices of atmospheric blocking and their statistical relationship with winter climate patterns in the Euro-Atlantic region. *International Journal of Climatology*, **26(2)**, 233–250 (2006).

Sha, L., et al. Solar forcing as an important trigger for West Greenland sea-ice variability over the last millennium. *Quaternary Science Reviews*, **131**, 148–156 (2016).

Sicre, M.-A., et al. Decadal variability of sea surface temperatures off North Iceland over the last 2000 years. *Earth and Planetary Science Letters*, **268(1)**, 137–142 (2008).

Spielhagen, R. F., et al. Enhanced modern heat transfer to the Arctic by warm Atlantic water. *Science*, **331(6016)**, 450–453 (2011).

Von Storch, H. and Zwiers, F. W. *Statistical Analysis In Climate Research.* Cambridge University press (2001).

Werner, K., et al. Atlantic Water advection to the eastern Fram Strait—Multiproxy evidence for late Holocene variability. *Palaeogeography, Palaeoclimatology, Palaeoecology*, **308(3)**, 264–276 (2011).
